# Phosphorylation of PNKP by ATM prevents its proteasomal degradation and enhances resistance to oxidative stress

**DOI:** 10.1093/nar/gks909

**Published:** 2012-10-05

**Authors:** Jason L. Parsons, Svetlana V. Khoronenkova, Irina I. Dianova, Nicola Ternette, Benedikt M. Kessler, Pran K. Datta, Grigory L. Dianov

**Affiliations:** ^1^Gray Institute for Radiation Oncology and Biology, Department of Oncology, University of Oxford, Roosevelt Drive, Oxford OX3 7DQ, UK, ^2^Nuffield Department of Clinical Medicine, University of Oxford, Roosevelt Drive, Oxford OX3 7BN, UK and ^3^The University of Alabama at Birmingham, Department of Medicine, Birmingham, AL 35294, USA

## Abstract

We examined the mechanism regulating the cellular levels of PNKP, the major kinase/phosphatase involved in the repair of oxidative DNA damage, and find that it is controlled by ATM phosphorylation and ubiquitylation-dependent proteasomal degradation. We discovered that ATM-dependent phosphorylation of PNKP at serines 114 and 126 in response to oxidative DNA damage inhibits ubiquitylation-dependent proteasomal degradation of PNKP, and consequently increases PNKP stability that is required for DNA repair. We have also purified a novel Cul4A-DDB1 ubiquitin ligase complex responsible for PNKP ubiquitylation and identify serine–threonine kinase receptor associated protein (STRAP) as the adaptor protein that provides specificity of the complex to PNKP. *Strap*^−/−^ mouse embryonic fibroblasts subsequently contain elevated cellular levels of PNKP, and show elevated resistance to oxidative DNA damage. These data demonstrate an important role for ATM and the Cul4A-DDB1-STRAP ubiquitin ligase in the regulation of the cellular levels of PNKP, and consequently in the repair of oxidative DNA damage.

## INTRODUCTION

Cellular oxidative metabolism and exogenous agents, such as ionizing radiation, generate DNA damage through the formation of reactive oxygen species (ROS) that attack DNA (1). ROS can generate oxidative DNA base damage, base loss (abasic site) and DNA single-strand breaks (SSBs) containing various modified 5′- and/or 3′-ends, such as 5′-hydroxyl, 3′-phosphoglycolate and 3′-phosphate ends (2). The majority of these DNA lesions are predominantly repaired by proteins of the base excision repair (BER) pathway [reviewed in (3)]. If the repair of oxidative base lesions by BER is initiated by OGG1 or NTH1, the damaged base is removed from the DNA by hydrolysis of the N-glycosylic bond linking the base to the sugar phosphate backbone and the resulting abasic site is recognized by AP endonuclease-1 that incises the phosphodiester bond to generate a DNA SSB containing 5′-deoxyribose phosphate and 3′-hydroxyl ends. DNA polymerase β consequently excises the 5′-deoxyribose phosphate moiety and adds an undamaged nucleotide into the DNA repair gap, which is subsequently ligated by XRCC1-DNA ligase IIIα complex (4). However, BER of oxidative lesions that is initiated by the endonuclease VIII-like (NEIL1-3) proteins, proceeds via an APE1-independent pathway (5) that requires the activity of polynucleotide kinase phosphatase (PNKP), as the action of NEIL glycosylases on DNA base damage generates a 1 nt gap containing 3′-phosphate ends that require further processing to generate 3′-hydroxyl termini for DNA polymerase β action.

PNKP is a 57 kDa dual-function enzyme containing both 5′-kinase and 3′-phosphatase activities for DNA ends (6,7). The importance of PNKP in the repair of oxidative DNA lesions has been demonstrated by the observation that a stable shRNA knockdown of PNKP in human cells sensitizes the cells to hydrogen peroxide, and also to ionizing radiation (8). Furthermore, mutations in the *pnkp* gene leading to significantly reduced PNKP protein levels have recently been found in patients with microcephaly, early-onset, intractable seizures and developmental delay (also termed MCSZ), an autosomal recessive disease characterized by severe neurological abnormalities. Cells derived from the patient’s lymphoblasts were observed to be significantly impaired in their ability to repair oxidative DNA damage (9). As a consequence of the reduced PNKP protein, and therefore activity, in these patients this should lead to an accumulation of unrepaired DNA SSBs. Furthermore, since activation of the protein kinase ATM plays a central role in the DNA damage response, this suggests that there should be a regulatory link between PNKP and ATM. Indeed, two groups have recently demonstrated that ATM activated by ionizing radiation-induced DNA damage phosphorylates PNKP at serines 114 and 126 and increases repair of DNA double-strand breaks (10,11). However the link between PNKP phosphorylation by ATM and the repair of oxidative DNA damage, as well as the mechanism involved in the increase in PNKP activity after phosphorylation, remains unclear.

Here we report that phosphorylation of PNKP by ATM in response to oxidative DNA damage prevents its ubiquitylation and subsequent degradation, thus leading to PNKP accumulation and an increased ability to repair the DNA damage. We subsequently identify a novel Cul4A-DDB1-STRAP protein complex as the major E3 ubiquitin ligase involved in PNKP ubiquitylation, which thus regulates the cellular steady-state levels of PNKP and cellular resistance to oxidative DNA damage.

## MATERIALS AND METHODS

### Materials

The cDNA for PNKP containing an N-terminal hexahistidine-tag cloned into pET28a was used for site-directed PCR mutagenesis to generate site-specific mutants using the QuikChange® Site-Directed Mutagenesis Kit (Agilent Technologies, Stockport, UK). Truncated versions of PNKP consisting of amino acids 1-130, 1-264 and 1-400 were prepared by PCR cloning. Recombinant proteins were expressed in *Escherichia coli* and purified using HisTrap HP and Mono-S HR 5/5 column chromatography (GE Healthcare, Little Chalfont, UK). The cDNA for PNKP was sub-cloned into pCMV-3Tag-3a vector (Agilent Technologies, Stockport, UK) for mammalian expression containing a C-terminal 3 × Flag-tag using the ligation-independent cloning (LIC) technique (12). Mammalian expression plasmids for HA-tagged Cul4A and Flag-tagged DDB1 were obtained from Addgene (Cambridge, USA) and that for HA-tagged ubiquitin was kindly provided by Prof. D. Bohmann. The mammalian expression plasmid for Flag-STRAP, as well as *strap*^+/+^ and *strap*^−^^/^^−^ MEFs are previously described (13,14). HA, DDB1 and ATM antibodies were purchased from Abcam (Cambridge, UK), Flag antibodies were from Agilent Technologies (Stockport, UK), Cul4A and phospho-PNKP (S114/T118) antibodies were from Cell Signalling Technology (Danvers, USA), STRAP (serine–threonine kinase receptor associated protein) and DNA-PKcs antibodies were from Santa Cruz Biotechnology (Santa Cruz, USA) and tubulin antibodies were from Sigma-Aldrich (Gillingham, UK). PNKP antibodies were kindly provided by Dr M. Weinfeld. Ubiquitin, E1 activating enzyme and E2 conjugating enzymes were purchased from Boston Biochemicals (Cambridge, USA).

### RNA interference and complementation

All cells were cultured as a monolayer in DMEM medium containing 10% FBS. For siRNA knockdowns and complementation studies, HCT116p53^+/+^ cells were grown in 10 cm dishes for 24 h to 30–50% confluence and then treated with 10 µl Lipofectamine RNAiMAX reagent (Invitrogen, Paisley, UK) in the presence of 200 pmol PNKP siRNA (5′-CACACUGUAUUUGGUCAAU-3′) for a further 48 h. Cells were then treated with 10 μl Lipofectamine transfection reagent (Invitrogen, Paisley, UK) in the presence of the siRNA-resistant mammalian expression plasmids (100 ng) for a further 24 h. The siRNA sequence used to knockdown ATM was 5′-AACATACTACTCAAAGACATT-3′, as previously described (15).

### Whole cell extracts

Whole cell extracts were prepared by Tanaka’s method (16). Briefly, cells were resuspended in one packed-cell volume of buffer containing 10 mM Tris-HCl (pH 7.8), 200 mM KCl, 1 mg/ml of each protease inhibitor (pepstatin, aprotinin, chymostatin and leupeptin), 1 mM PMSF and 1 mM NEM. Two packed-cell volumes of buffer containing 10 mM Tris-HCl (pH 7.8), 600 mM KCl, 40% glycerol, 0.1 mM EDTA and 0.2% Nonidet P-40 was then added and mixed thoroughly before rocking the cell suspension for 2 h at 4°C. The cell lysate was then centrifuged at 40 000 rpm at 4°C for 20 min and the supernatant was collected, aliquoted and stored at −80°C.

### Western blotting

Western blots were performed by standard procedure as recommended by the vendor (Novex, San Diego, USA). Blots were visualized using the Odyssey image analysis system (Li-cor Biosciences, Cambridge, UK).

### Purification of the PNKP E3 ubiquitin ligase from HeLa whole cell extracts

Whole cell extracts were prepared from 20 g HeLa cell pellets (Cilbiotech, Mons, Belgium) by Tanaka’s method (16) and dialysed against Buffer A (50 mM Tris–HCl (pH 8.0), 1 mM EDTA, 5 % glycerol, 1 mM DTT and 1 mM PMSF) containing 150 mM KCl. The extract (1 g) was then applied to a 100 ml Phosphocellulose column and the flow-through collected (PC-FI). PC-FI was diluted 2-fold to achieve a final concentration of 75 mM KCl in the fraction and then added to a 20 ml HiLoad Mono-Q Sepharose column (GE Healthcare, Little Chalfont, UK), washed with Buffer A containing 50 mM KCl and proteins eluted using a linear gradient from 50–1000 mM KCl. Active fractions were pooled, concentrated using Amicon Ultra-15 filter units (Millipore, Watford, UK) and loaded onto a Superdex 200 HR 10/30 column (GE Healthcare, Little Chalfont, UK) in Buffer A containing 150 mM KCl and 0.5 ml fractions collected. Active fractions were pooled, concentrated using Amicon Ultra-4 filter units (Millipore, Watford, UK) and dialysed against Buffer B containing 5 mM potassium phosphate (pH 7.0), 5% glycerol, 1 mM DTT and 1 mM PMSF. The sample was added to a 1 ml CHT-hydroxyapatite column (Bio-Rad, Hemel Hempstead, UK) in Buffer B and proteins were eluted using a linear gradient of 5–500 mM potassium phosphate. Active fractions were pooled, concentrated using Amicon Ultra-4 filter units (Millipore, Watford, UK) and then loaded onto a Mini-Q PC 3.2/3 column (GE Healthcare, Little Chalfont, UK) in buffer A containing 50 mM KCl. Proteins were eluted using a linear gradient of 50–1000 mM KCl and 50 µl fractions collected. At each chromatography stage, aliquots of the fractions were analysed for E3 ubiquitin ligase activity using PNKP as a substrate and active fractions pooled for the next chromatography step. Active fractions from the final Mini-Q chromatography stage were analysed by mass spectrometry to identify candidate E3 ubiquitin ligases.

### *In vitro* ubiquitylation assay

Assays were performed in a 15 µl reaction volume in the presence of 3.5 pmol PNKP, 0.7 pmol E1 activating enzyme, 6 pmol E2 conjugating enzyme and 0.6 nmol ubiquitin (Boston Biochemicals, Cambridge, USA) in buffer containing 25 mM Tris–HCl (pH 8.0), 4 mM ATP, 5 mM MgCl_2_, 200 µM CaCl_2_, 1 mM DTT, 10 µM MG-132 for 1 h at 30°C. SDS–PAGE sample buffer (25 mM Tris–HCl (pH 6.8), 2.5% β-mercaptoethanol, 1% SDS, 5% glycerol, 1 mM EDTA, 0.15 mg/ml bromophenol blue) was added, the samples were heated for 5 min at 95°C prior to separation of the proteins on a 10% SDS-polyacrylamide gel, followed by transfer to a PVDF membrane and immunoblot analysis with PNKP antibodies.

### *In vivo* ubiquitylation of PNKP

*Strap*^+/+^ and *strap*^−^^/^^−^ MEFs were grown in 10 cm dishes for 24 h to 80–90% confluency and then treated with 10 µl Lipofectamine transfection reagent (Invitrogen, Paisley, UK) in the presence of mammalian expression plasmids for HA-tagged ubiquitin (1 µg) and Flag-tagged PNKP (1 µg) for a further 24 h. Alternatively, HCT116p53^+/+^ cells were treated with PNKP siRNA (200 pmol), in the absence and presence of ATM siRNA (200 pmol), and transfected with an siRNA-resistant mammalian expression plasmid for Flag-tagged PNKP (100 ng), as described in the section above, but also in the presence of HA-tagged ubiquitin (1 µg). Cells were subsequently incubated with the proteasomal inhibitor MG-132 (10 µM) for 6 h, pelleted by centrifugation, whole cell extracts were prepared and equivalent amounts of protein in the extracts were incubated with 10 µl anti-Flag magnetic beads (Sigma-Aldrich, Gillingham, UK) for 2 h at 4°C with rotation. The beads were separated from the extract using a magnetic separation rack and washed 3 times with 500 µl buffer containing 50 mM Tris–HCl (pH 8.0), 150 mM KCl, 1 mM EDTA and 5% glycerol prior to the addition of SDS-PAGE sample buffer. The samples were heated for 5 min at 95°C prior to separation of the proteins on a 10% SDS-polyacrylamide gel, followed by transfer to a PVDF membrane and immunoblot analysis with HA antibodies to determine the degree of PNKP ubiquitylation.

### RT-PCR analysis

RNA was prepared from *strap*^+/+^ and *strap*^−^^/^^−^ MEFs using the RNeasy kit (Qiagen, Crawley, UK) and cDNA was subsequently generated using the SuperScript RT-PCR system (Invitrogen, Paisley, UK). PNKP and actin was amplified using the following primers: 5′-GTTACTGGTGTTCACAGCGTCT-3′ and 5′-GATGAGGGTCCCATCTAGGTC-3′, 5′-GGAGGGGGTTGAGGTGTT-3′ and 5′-TGTGCACTTTTATTGGTCTCAAG-3′, respectively. Cycling conditions of 1 cycle at 95°C for 5 min, 25 cycles at 95°C for 30 s, 55°C for 20 s and 72°C for 20 s and 1 cycle at 72°C for 5 min were used. PCR products were separated by 2% agarose gel electrophoresis containing SYBR Green (Invitrogen, Paisley, UK) and analysed using the Molecular Imager FX system (Bio-Rad, Hemel Hempstead, UK).

### Cell survival assay

Cells were treated with up to 800 µM hydrogen peroxide for 15 min in DMEM medium. Colonies that appeared after ∼10 days were fixed with methanol, stained with Methylene Blue and counted. Relative colony formation (% survival) was expressed as colonies per treatment level versus colonies that appeared in the control.

### Protein identification and mapping of ubiquitylation sites by tandem mass spectrometry

Proteins in chromatography fractions were precipitated using chloroform and methanol as described previously (17), followed by in-solution trypsin digestion_._ Digested material was desalted using C18 Sep-Pack cartridges (Waters, Milford, USA) according to the manufacturer’s instructions and concentrated by vacuum centrifugation. Digested material was analysed by LC-MS/MS tandem mass spectrometry using either a high capacity iontrap (HCTplus™, Bruker Daltonics, Bremen, Germany) as described (18) or a nanoAcquity UPLC system coupled to a quadrupole time-of-flight (QTOFpremier™) tandem mass spectrometer (Waters, Milford, USA). The interpretation and presentation of MS/MS data was performed according to published guidelines (19). MS/MS spectra (peaklists) were searched against the SwissProt database (release version 54.0, 07/2007, number of entries 276256) using Mascot version 2.2 (Matrixscience, London, UK). In addition, individual MS/MS spectra for peptides with a Mascot Mowse score lower than 40 (Expect <0.015) were inspected manually and included in the statistics only if a series of at least 4 continuous y or b-ions were observed. The local ‘in-house’ Mascot server used for this study is supported and maintained by the Computational Biology Research Group at the University of Oxford.

### Alkaline single cell gel electrophoresis (Comet) assay

The comet assay was performed as recently described (20,21). Briefly, cells were trypsinized, treated or mock-treated in suspension with hydrogen peroxide (35 µM) for 5 min on ice and embedded on a microscope slide in agarose (Bio-Rad, Hemel Hempstead, UK). The slides were incubated for various times at 37°C in a humidified chamber to allow for DNA repair, prior to lysis in buffer containing 2.5 M NaCl, 100 mM EDTA, 10 mM Tris–HCl pH 10.5, 1% (v/v) DMSO and 1% (v/v) Triton X-100 for 1 h at 4°C. The slides were then incubated in the dark for 30 min in cold electrophoresis buffer (300 mM NaOH, 1 mM EDTA, 1% (v/v) DMSO, pH 13) to allow the DNA to unwind prior to electrophoresis at 25 V, 300 mA for 25 min. After neutralization with 0.5 M Tris–HCl (pH 8.0), the slides were stained with SYBR Gold (Invitrogen, Paisley, UK) and analysed using the Komet 5.5 image analysis software (Andor Technology, Belfast, Northern Ireland).

## RESULTS

### ATM-dependent phosphorylation regulates PNKP stability

**Figure 1.**
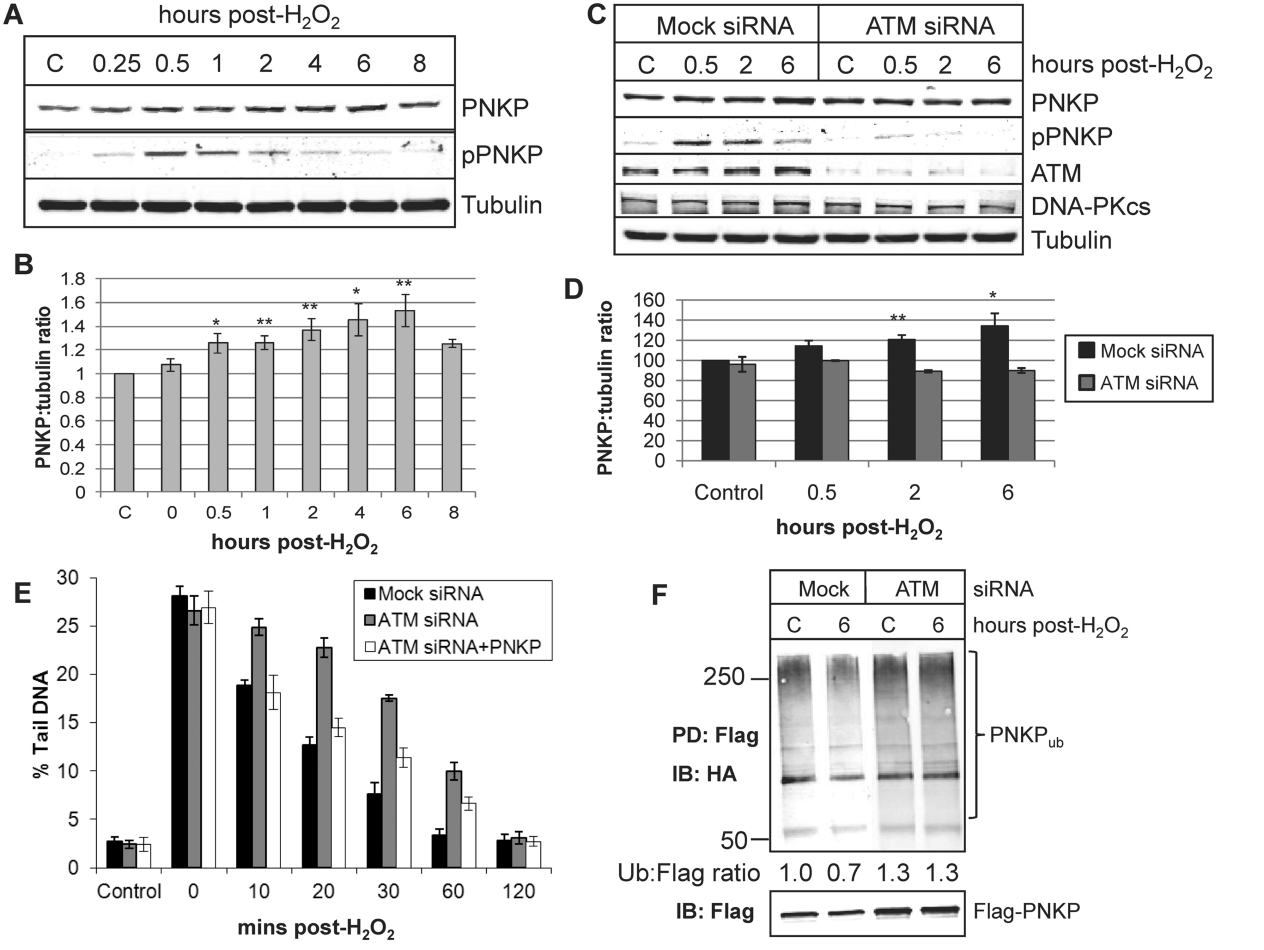
Phosphorylation-dependent regulation of PNKP protein levels in response to oxidative DNA damage. (**A**) HCT116p53^+/+^ cells were either untreated (C) or treated with 150 µM hydrogen peroxide and allowed to repair at 37°C for the time points indicated prior to harvesting. Whole cell extracts were prepared and analysed by 10% SDS-PAGE and immunoblotting with the indicated antibodies. (**B**) The protein levels of PNKP following hydrogen peroxide treatment were quantified and normalized against the protein levels of tubulin from at least three independent experiments. Shown is the mean PNKP:tubulin ratio with standard errors normalized to the control untreated cells which was set to 1.0. *P < 0.02, **P < 0.01 as analysed by Students *t*-test. (**C**) HCT116p53^+/+^ cells were grown in 10 cm dishes for 24 h to 30–50% confluency and then treated with Lipofectamine transfection reagent (10 µl) in the absence (Mock siRNA) or presence of 200 pmol ATM siRNA for 48 h and then either untreated (C) or treated with 150 µM hydrogen peroxide and allowed to repair at 37°C for the times indicated prior to harvesting. Whole cell extracts were prepared and analysed by 10% SDS-PAGE and immunoblotting with the indicated antibodies. (**D**) The protein levels of PNKP following Mock siRNA (black bars) or ATM siRNA (shaded bars) and subsequent hydrogen peroxide treatment were quantified and normalized against the protein levels of tubulin from at least three independent experiments. Shown is the mean PNKP:tubulin ratio with standard errors normalized to the Mock siRNA-control untreated cells which was set to 1.0. *P < 0.03, **P < 0.01 as analysed by Student's *t*-test. (**E**) HCT116p53^+/+^ cells were grown in 10 cm dishes for 24 h to 30–50% confluency and then treated with Lipofectamine transfection reagent (10 µl) in the absence (Mock siRNA) or presence of 200 pmol ATM siRNA for 24 h. Cells were then treated with Lipofectamine transfection reagent (10 µl) in the absence (Mock siRNA, black bars and ATM siRNA, shaded bars) and presence (ATM siRNA + PNKP, white bars) of a mammalian expression plasmid expressing Flag-tagged PNKP (500 ng) for a further 24 h. Cells were treated in suspension with 35 µM hydrogen peroxide, for 5 min, embedded in agarose on microscope slides and allowed to repair for the times indicated. The levels of DNA strand breaks and alkali-labile sites were then analysed by the alkaline single cell gel electrophoresis (comet) assay. Shown are the mean % tail DNA values with standard deviations from at least three independent experiments. (**F**) HCT116p53^+/+^ cells were grown in 10 cm dishes for 24 h to 30–50% confluency and then treated with Lipofectamine transfection reagent (10 µl) in the presence of 200 pmol PNKP siRNA (Mock) or 200 pmol PNKP and ATM siRNA for 24 h, followed by a further treatment with Lipofectamine transfection reagent (10 µl) in the presence of mammalian expression plasmids expressing siRNA-resistant Flag-tagged PNKP (100 ng) and HA-tagged ubiquitin (1 µg) for a further 24 h. Cells were then either untreated (C) or treated with 150 µM hydrogen peroxide for 15 min, followed by incubation with 10 µM MG-132 for 6 h. Whole cell extracts were prepared and incubated with anti-Flag magnetic beads for 2 h at 4°C with rotation. The beads were separated from the extract using a magnetic separation rack, washed several times with buffer containing 150 mM KCl prior to the addition of SDS loading dye and analysis by 10% SDS-PAGE and immunoblotting with HA or Flag antibodies. The ratio of expression levels of ubiquitylated PNKP compared to the unmodified protein, as determined using Flag antibodies, were calculated from at least three independent experiments and normalized to the levels observed in the Mock, untreated sample which was set to 1.0.

It has been recently described that phosphorylation of PNKP at serine 114 and 126, predominantly by the protein kinase ATM, occurs in response to ionizing radiation and is required for effective DNA double-strand break repair (10,11). To examine whether PNKP phosphorylation is induced by oxidative stress, we treated HCT116p53^+/+^ cells with 150 µM hydrogen peroxide and observed that PNKP phosphorylation at serine 114 is initiated within 15 min post-treatment, peaks at ∼30 min post-treatment and subsequently reaches baseline levels at between 6 and 8 h post-treatment (Figure 1A). This profile of PNKP phosphorylation correlates with previously published data on PNKP phosphorylation in response to ionizing radiation and oxidative DNA damage (11). Concurrent with the induction of PNKP phosphorylation in response to oxidative DNA damage, we also observed a moderate (about 1.5-fold at the peak), but highly reproducible, time-dependent increase in the protein levels of PNKP which reached a maximum at between 4 h and 6 h post-treatment (Figure 1A and B). This suggested that ATM-dependent phosphorylation of PNKP in response to DNA damage causes an increased stability in the protein. Indeed, we were able to show that an siRNA knockdown of ATM, which significantly reduces the level of PNKP phosphorylation, prevented the increased stability of PNKP in response to oxidative DNA damage (Figure 1C and D). It is important to note that the phosphospecific antibody used in this study detects PNKP phosphorylated at serine 114 and threonine 118. However since we, and others using antibodies specifically targeting PNKP phosphorylated at serine 114 (10,11), have shown that phosphorylation in response to DNA damage is ATM dependent, this suggests that these antibodies are most likely specific to phosphorylated serine 114. To demonstrate that this process is required for efficient DNA repair, we analysed the rate of repair of hydrogen peroxide-induced DNA strand breaks and alkali-labile sites by the comet assay. We observed that a delay in the rate of DNA repair observed in ATM siRNA knockdown cells versus control cells (Figure 1E; compare black and grey bars), can be mainly restored by transfection of ATM siRNA knockdown cells with a mammalian expression plasmid for PNKP (Figure 1E; compare grey and white bars; and Supplementary Figure S1). This suggests that ATM-dependent phosphorylation of PNKP, giving rise to increased protein stability, is required for efficient DNA repair in response to oxidative DNA damage. We hypothesized that the cellular protein levels of PNKP are regulated by the ubiquitin proteasome pathway, and that ubiquitylation of PNKP is modulated by ATM-dependent phosphorylation following oxidative stress. Indeed, we were able to show that PNKP is less efficiently ubiquitylated (reduced by ∼30%) following 6 h post-treatment with hydrogen peroxide, at a time where the amount of PNKP protein stability is maximal (Figure 1A and B), in comparison to untreated cells (Figure 1F; compare lanes 1 and 2). In contrast, the level of PNKP ubiquitylation following an ATM siRNA knockdown was unchanged in response to oxidative DNA damage (Figure 1F; compare lanes 3 and 4). Interestingly there was an elevated level of PNKP ubiquitylation, as measured by the ratio of ubiquitin to PNKP protein, in ATM siRNA knockdown cells than in control cells (Figure 1F; compare lanes 1 and 3), suggesting that ATM may also phosphorylate and control PNKP ubiquitylation in response to endogenous DNA damage.

### Purification of the cellular E3 ubiquitin ligase activity specific for PNKP

**Figure 2.**
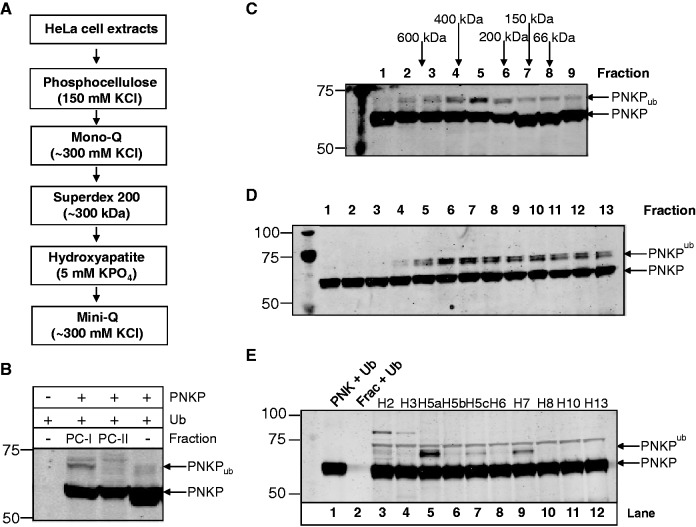
Purification of the E3 ubiquitin ligase for PNKP. (**A**) Purification scheme for the E3 ubiquitin ligase for PNKP from HeLa cell extracts. (**B**) *In vitro* ubiquitylation of His-tagged PNKP (3.5 pmol) by low salt elution (PC-I, 150 mM KCl) and high salt elution (PC-II; 1 M KCl) fractions (10 µg) obtained from Phosphocellulose chromatography. (**C**) *In vitro* ubiquitylation of His-tagged PNKP (3.5 pmol) by fractions obtained from Superdex 200 chromatography. (**D**) *In vitro* ubiquitylation of His-tagged PNKP (3.5 pmol) by fractions obtained from Mini-Q chromatography. (**E**) *In vitro* ubiquitylation of His-tagged PNKP (3.5 pmol) by active fraction (Frac) purified from HeLa whole cell extracts in the presence of various E2 conjugating enzymes. In all experiments, *in vitro* ubiquitylation of His-tagged PNKP (3.5 pmol) was performed in the presence of E1 activating enzyme (0.7 pmol), ubiquitin (0.6 nmol; Ub) and all E2 conjugating enzymes (6 pmol each), except in (E) where individual E2 enzymes were used, and analysed by 10% SDS-PAGE and immunoblotting using PNKP antibodies. Molecular weight markers are indicated on the left-hand side of appropriate figures and the positions of ubiquitylated PNKP (PNKP_ub_) are shown.

Since we identified that ubiquitylation of PNKP is modulated by ATM-dependent phosphorylation in response to oxidative DNA damage, we therefore proceeded to purify the major human E3 ubiquitin ligase activity involved in PNKP ubiquitylation. We fractionated HeLa cell extracts over a series of chromatography columns and monitored fractions for *in vitro* ubiquitylation activity using PNKP as a substrate (Figure 2A). We observed that ubiquitylation activity against PNKP could be detected after Phosphocellulose chromatography, predominantly in the low salt (150 mM KCl) elution fraction PC-I (Figure 2B) and following size-exclusion chromatography the ubiquitin ligase was found to elute with a molecular weight of between 200 kDa and 400 kDa (Figure 2C). Interestingly, the activity appeared to predominantly result in monoubiquitylation of PNKP (observed molecular weight of <70 kDa equivalent to the size of PNKP (57 kDa) and a single ubiquitin molecule (8 kDa)) and this was further demonstrated by the fractions from the final Mini-Q chromatography column (Figure 2D), which displayed robust monoubiquitylation activity against PNKP. This ubiquitylation activity against PNKP was not observed in the absence of active fraction or in the absence of PNKP in the activity reaction mixture (Figure 2E, lanes 1 and 2, respectively), indicating specificity of ubiquitylation. We were also able to demonstrate a preference of the ubiquitylation activity for the H5a E2 conjugating enzyme, and to a lesser extent with the H5c and H7 E2 conjugating enzymes (Figure 2E, lanes 5, 7 and 9, respectively).

### Identification of the PNKP E3 ubiquitin ligase purified from human cells

**Figure 3.**
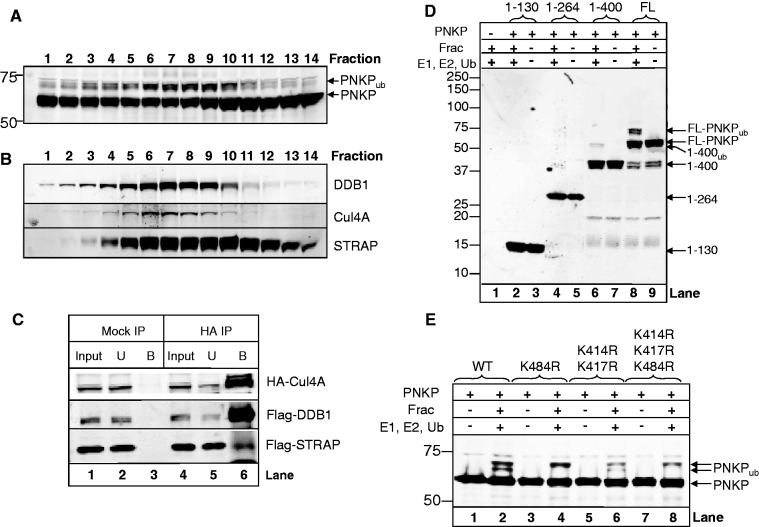
Identification of Cul4A-DDB1-STRAP as the E3 ubiquitin ligase for PNKP and identification of the major ubiquitylation sites. (**A**) *In vitro* ubiquitylation of PNKP (3.5 pmol) by fractions obtained from Superdex 200 chromatography purified from HeLa whole cell extracts in the presence of E1 activating enzyme (0.7 pmol), H5a E2 conjugating enzyme (6 pmol) and ubiquitin (0.6 nmol) analysed by 10% SDS-PAGE and immunoblotting using PNKP antibodies. (**B**) Fractions were also analysed by 10% SDS-PAGE and immunoblotting using Cul4A, DDB1 and STRAP antibodies. (**C**) HeLa cells were grown in 10 cm dishes for 24 h to 80–90% confluency and then treated with Lipofectamine (10 µl) in the presence of mammalian expression plasmids for HA-tagged Cul4A (1.5 µg), Flag-tagged DDB1 (1.5 µg) and Flag-tagged STRAP (1.5 µg) for 24 h. Whole cell extracts were prepared and incubated with either magnetics beads (Mock IP) or anti-HA magnetic beads (HA IP) for 2 h at 4°C with rotation. The beads were separated from the extract using a magnetic separation rack, washed several times with buffer containing 150 mM KCl prior to the addition of SDS loading dye and analysis by 10% SDS-PAGE and immunoblotting with HA or Flag antibodies. The input (5%), unbound (U) and proteins bound to the magnetic beads (B) are shown. (**D**) *In vitro* ubiquitylation of full-length PNKP (FL) and PNKP truncated protein fragments (3.5 pmol) by active fraction (Frac) containing Cul4A-DDB1-STRAP purified from HeLa whole cell extracts in the presence of E1 activating enzyme (0.7 pmol), H5a E2 conjugating enzyme (6 pmol) and ubiquitin (0.6 nmol) were analysed by 4–20% SDS-PAGE and immunoblotting using PNKP antibodies. (**E**) Recombinant wild type PNKP (WT) or PNKP mutants (3.5 pmol) were *in vitro* ubiquitylated using active fraction (Frac) purified from HeLa whole cell extracts in the presence of E1 activating enzyme (0.7 pmol), H5a E2 conjugating enzyme (6 pmol) and ubiquitin (0.6 nmol) and analysed by 10% SDS-PAGE and immunoblotting using PNKP antibodies. Molecular weight markers are indicated on the left-hand side of appropriate figures and the positions of ubiquitylated PNKP (PNKP_ub_) and ubiquitylated 1-400 amino acid fragment of PNKP (1-400_ub_) are shown.

Fractions from the final Mini-Q chromatography column (Figure 2D) were analysed by nanoLC-MS/MS tandem mass spectrometry which identified the Cullin 4A scaffold protein (Cul4A; Mascot score: 395, 26% sequence coverage; Supplementary Figure S2A) and DNA damage binding protein-1 (DDB1; Mascot score: 175, 21% sequence coverage; Supplementary Figure S2B). Active fractions purified from HeLa cell extracts from the size-exclusion chromatography column stage demonstrating robust PNKP ubiquitylation activity (Figure 3A) were analysed by western blotting using Cul4A and DDB1 antibodies. Both Cul4A and DDB1 proteins in the fractions aligned very well with the purified PNKP ubiquitylation activity (Figure 3B), most likely demonstrating that they form the E3 ubiquitin ligase complex that is ubiquitylating PNKP. It has been previously shown that Cul4A-DDB1-Roc1 form a core ubiquitin ligase complex and that substrate specificity is provided by interaction of DDB1 with a WD-40 repeat protein that specifically interacts with the protein which is targeted for ubiquitylation (22–25). We identified the only WD-40 repeat protein in our purified active fractions by mass spectrometry as serine–threonine kinase receptor-associated protein (STRAP; Mascot score: 750, 49% sequence coverage; Supplementary Figure S2C) which was subsequently found to correlate very well with the distribution of both Cul4A/DDB1 proteins and PNKP ubiquitylation activity observed following size-exclusion chromatography (Figure 3B). We also found that the STRAP protein sequence aligned well with the conserved tandem repeat consensus sequence of DXXXR/KXWDXR/K, also known as the DWD (DDB1-binding WD-40 protein) box, which has been shown to bind DDB1 and found in known DDB1-interacting proteins such as CDT2, DDB2 and CSA (reviewed in (26); Supplementary Figure S3). To prove the existence of the Cul4A-DDB1-STRAP ubiquitin ligase complex in living cells, we simultaneously expressed HA-tagged Cul4A, Flag-tagged DDB1 and Flag-tagged STRAP in HeLa cells and then performed an immunoprecipitation using anti-HA antibodies bound to magnetic beads (Figure 3C). We demonstrated that neither Cul4A, DDB1 nor STRAP bind non-specifically to the agarose beads when a mock pulldown was performed (Figure 3C, lane 3). In contrast, an anti-HA antibody pulldown confirmed the interaction of Cul4A with DDB1, but also revealed the presence of STRAP in the immunoprecipitate (Figure 3C, lane 6). These data demonstrate that the ubiquitin ligase complex for PNKP purified from HeLa cell extracts is Cul4A-DDB1-STRAP.

### Identification of the ubiquitylation sites within PNKP

The PNKP protein consists of three major domains, namely the N-terminal forkhead-associated (FHA) domain, a central phosphatase domain and a C-terminal kinase domain (Supplementary Figure S4A). To determine the sites of ubiquitylation, we generated three truncations of PNKP, namely amino acids 1-130 (containing the FHA domain), 1-264 (containing the FHA and part of the phosphatase domain) and 1-400 (containing the FHA, phosphatase and part of the kinase domain; Supplementary Figure S4B). We subsequently analysed the *in vitro* ubiquitylation of these PNKP truncations, along with the full-length protein, using an active fraction purified from HeLa cell extracts known to contain the Cul4A-DDB1-STRAP ubiquitin ligase complex. As previously shown, we observed efficient monoubiquitylation of full-length PNKP, and in fact this appeared as a doublet, indicating potentially two amino acid sites of modification (Figure 3D, lane 8). In contrast, there was a significantly reduced amount of ubiquitylation of the 1-400 truncation (Figure 3D, lane 6) and almost no ubiquitylation of either the 1-264 (Figure 3D, lane 4) or 1-130 truncations (Figure 3D, lane 2). These data suggested that the majority of ubiquitylation of PNKP by Cul4A-DDB1-STRAP was occurring within the kinase domain of the protein, and specifically within amino acids 401-521 of the protein. Since ubiquitylation of proteins commonly occurs on lysine residues, we examined the number of lysine residues within the kinase domain of PNKP (400-521) and discovered that only three lysines were present in this region, namely lysines 414, 417 and 484. To discover the major sites of ubiquitylation, we mutated either lysines 414 and 417 together or lysine 484 to arginine and then analysed *in vitro* ubiquitylation using an active fraction containing Cul4A-DDB1-STRAP. We found that when lysine 484 was mutated to arginine, there was a significant reduction in ubiquitylation of PNKP, in particular the lower band of the doublet of monoubiquitylation disappeared in contrast to the wild-type (WT) protein (Figure 3E, compare lanes 2 and 4). Furthermore, the lysine 414/417 double mutant showed a significant reduction (∼60%) in both bands of the doublet of monoubiquitylation compared to the WT protein (Figure 3E, compare lanes 2 and 6) and this reduction was further enhanced (∼70% reduction) when lysine 484 was additionally mutated (Figure 3E, lane 8). This demonstrates that all three lysine residues (414, 417 and 484) are the major sites of ubiquitylation within PNKP *in vitro*.

### Ubiquitylation-dependent degradation of PNKP is inhibited by ATM-dependent phosphorylation

**Figure 4.**
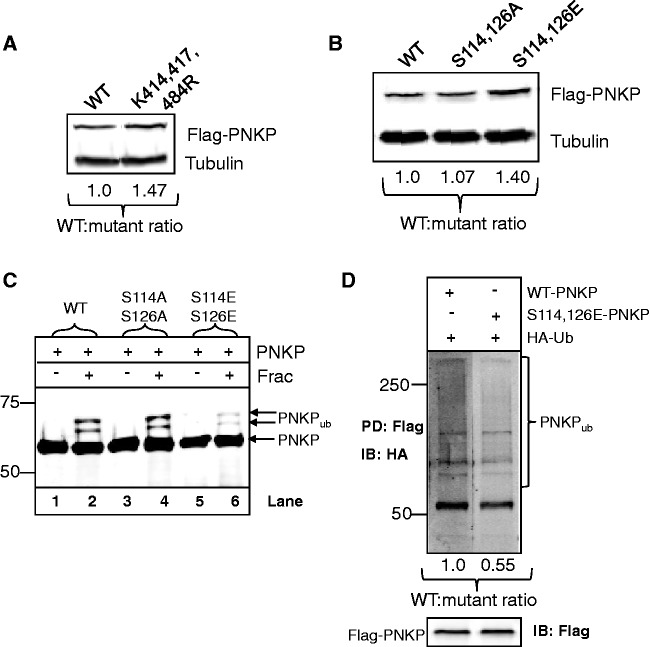
Phosphorylation of PNKP by ATM modulates its ubiquitylation-dependent degradation. (**A** and **B**) HCT116p53^+/+^ cells were grown in 10 cm dishes for 24 h to 30–50% confluency and then treated with Lipofectamine transfection reagent (10 µl) in the presence of 200 pmol PNKP siRNA for 48 h, followed by a further treatment with Lipofectamine transfection reagent (10 µl) in the presence of a mammalian expression plasmid expressing siRNA-resistant Flag-tagged WT, K414/417/484R triple mutant PNKP or S114/126A or S114/126E double mutant PNKP (100 ng) for a further 24 h. Cells were pelleted by centrifugation, whole cell extracts were prepared and analysed by 10% SDS-PAGE and immunoblotting with the indicated antibodies. The ratio of expression levels of mutant proteins compared to the WT protein, as determined using Flag antibodies and normalized against the tubulin loading control, were calculated from at least three independent experiments. (**C**) Recombinant WT PNKP (WT) or S114/126A or S114/126E double PNKP mutants (3.5 pmol) were *in vitro* ubiquitylated using active fraction containing Cul4A-DDB1-STRAP purified from HeLa whole cell extracts in the presence of E1 activating enzyme (0.7 pmol), H5a E2 conjugating enzyme (6 pmol) and ubiquitin (0.6 nmol) and analysed by 10% SDS-PAGE and immunoblotting using PNKP antibodies. (**D**) HCT116p53^+/+^ cells were grown in 10 cm dishes for 24 h to 30–50% confluency and then treated with Lipofectamine (10 µl) in the presence of 200 pmol PNKP siRNA for 48 h, followed by a further treatment with Lipofectamine transfection reagent (10 µl) in the presence of a mammalian expression plasmid expressing siRNA-resistant Flag-tagged WT or S114/126E double mutant PNK (100 ng) for a further 24 h. Following incubation of the cells with MG-132 (10 µM) for 6 h, the cells were pelleted by centrifugation, whole cell extracts were prepared and incubated with anti-Flag magnetic beads (10 µl) for 2 h at 4°C with rotation. The beads were separated from the extract using a magnetic separation rack, washed several times with buffer containing 150 mM KCl prior to the addition of SDS loading dye and analysis by 10% SDS-PAGE and immunoblotting with HA (upper panel) or Flag (lower panel) antibodies. The ratio of expression levels of ubiquitylated PNKP compared to the unmodified protein, as determined using Flag antibodies, were calculated from at least three independent experiments and normalized to the levels observed with the WT protein which was set to 1.0. Molecular weight markers are indicated on the left-hand side of appropriate figures and the positions of ubiquitylated PNKP (PNKP_ub_) are shown.

The most common consequence of cellular ubiquitylation of proteins is to target the proteins for degradation by the 26S proteasome. Therefore, to analyse whether lysines 414, 417 and 484 are promoting ubiquitylation-dependent degradation of PNKP *in vivo*, we firstly down-regulated endogenous PNKP by siRNA and then transfected cells with mammalian expression plasmids encoding WT or the ubiquitylation-deficient K414/417/484 R triple mutant PNKP, each of which also contained silent mutations to ensure resistance against the siRNA. Even though *in vitro* ubiquitylation was not fully ablated (Figure 3E), the triple mutant of PNKP was found to be almost 1.5-fold more stable than the WT protein, indicating that lysines 414, 417 and 484 are the major sites that promote PNKP ubiquitylation and degradation *in vivo* (Figure 4A). Since we demonstrated that ATM-dependent phosphorylation of PNKP in response to oxidative DNA damage prevents its ubiquitylation resulting in an increase in the cellular protein levels of PNKP (Figure 1C and D), we analysed this further by generating site-specific mutants of PNKP at serines 114 and 126, the major ATM-dependent phosphorylation sites. We created a serine to alanine PNKP double mutant that is unable to be phosphorylated (S114/126A) as well as a serine to glutamic acid PNKP mutant that mimics ATM-dependent phosphorylation (S114/126E). We show that when the S114/126A PNKP mutant is transfected into HCT116p53^+/+^ cells, the protein has a similar level of stability compared to the WT protein, whereas the phosphomimetic S114/126E protein was ∼1.4-fold more stable (Figure 4B). We also find that the S114/126E phosphomimetic double mutant of PNKP is less susceptible (∼50% reduction) to *in vitro* ubiquitylation by purified Cul4A-DDB1-STRAP, in comparison to the WT protein (Figure 4C, compare lanes 2 and 6). In contrast, the S114/126A mutant is ubiquitylated as efficiently as the WT protein *in vitro* (Figure 4C, compare lanes 2 and 4). We also found that the S114/126E phosphomimetic double mutant of PNKP was less efficiently ubiquitylated *in vivo* (approximate reduction of 45%) following transfection into HCT116p53^+/+^ cells, in comparison to the WT protein (Figure 4D). Cumulatively, these data demonstrate that ATM-dependent phosphorylation of PNKP at serines 114 and 126 promotes protein stability by inhibiting ubiquitylation-dependent proteasomal degradation.

### STRAP regulates the cellular protein levels of PNKP and modulates sensitivity to oxidative stress

**Figure 5.**
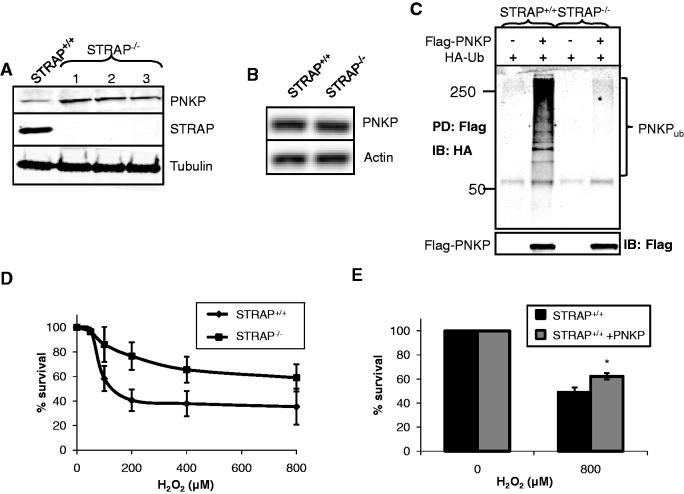
Cul4A-DDB1-STRAP regulates the cellular level of PNKP via ubiquitylation-dependent degradation and modulates cellular resistance to oxidative DNA damage. (**A**) Whole cell extracts were prepared from *strap*^+/+^ and three independent *strap*^−/−^ MEFs and were analysed by 10% SDS-PAGE and immunoblotting with the indicated antibodies. (**B**) cDNA was prepared from RNA from *strap*^+/+^ and *strap*^−/−^ MEFs and the levels of *pnkp* and *actin* mRNA were analysed by RT-PCR. (**C**) *Strap*^+/+^ and *strap*^−/−^ MEFs were grown in 10 cm dishes for 24 h to 80–90% confluency and then treated with Lipofectamine (10 µl) in the presence of mammalian expression plasmids for HA-tagged ubiquitin (1 µg) and Flag-tagged PNKP (1 µg) for a further 24 h. Following incubation of the cells with MG-132 (10 µM) for 6 h, the cells were pelleted by centrifugation, whole cell extracts were prepared and incubated with anti-Flag magnetic beads (10 µl) for 2 h at 4°C with rotation. The beads were separated from the extract using a magnetic separation rack, washed several times with buffer containing 150 mM KCl prior to the addition of SDS loading dye and analysis by 10% SDS-PAGE and immunoblotting with HA (upper panel) or Flag (lower panel) antibodies. (**D**) Clonogenic survival of *strap*^+/+^ and *strap*^−/−^ MEFs was analysed following treatment with increasing doses of hydrogen peroxide (0–800 µM). Shown is the % survival with standard deviations from at least three independent experiments. (**E**) *Strap*^+/+^ MEFs were grown in 10 cm dishes for 24 h to 80–90% confluency and then treated with Lipofectamine (10 µl) in the absence (black bars) and presence (shaded bars) of a mammalian expression plasmid for Flag-tagged PNKP (1 µg) for a further 24 h. Clonogenic survival of the cells following treatment with 0 and 800 µM hydrogen peroxide was analysed and shown is the % survival with standard deviations from at least three independent experiments. *P < 0.01 as analysed by Student's *t*-test.

Although we demonstrated that PNKP is targeted for ubiquitylation-dependent proteasomal degradation, the role of the E3 ubiquitin ligase Cul4A-DDB1-STRAP in this process *in vivo* remained to be shown. To demonstrate this, we analysed the protein levels of PNKP in *strap*^+/+^ and *strap*^−^^/^^−^ mouse embryonic fibroblasts (MEFs). As expected, we observed a ∼3–4-fold increase in PNKP protein levels in three independent *strap*^−^^/^^−^ MEFs, compared to *strap*^+/+^ MEFs (Figure 5A). To ensure that the increased protein levels of PNKP were as a consequence of the regulation of PNKP at the protein level, rather than as a consequence of increased DNA transcription, we analysed the levels of *pnkp* mRNA by RT-PCR and observed that equal amounts exist in *strap*^+/+^ and *strap*^−^^/^^−^ MEFs (Figure 5B). Furthermore, when we immunoprecipitated PNKP from *strap*^+/+^ and *strap*^−^^/^^−^ MEFs co-transfected with mammalian expression plasmids encoding Flag-tagged PNKP and HA-tagged ubiquitin, we observed that *strap*^+/+^ MEFs contain a significantly higher level of PNKP ubiquitylation activity (Figure 5C, compare lanes 2 and 4). This demonstrates that the Cul4A-DDB1-STRAP ubiquitin ligase complex is polyubiquitylating PNKP in mammalian cells. Finally to demonstrate the role of Cul4A-DDB1-STRAP in the cellular response to DNA damage, we compared clonogenic survival of *strap*^+/+^ and *strap*^−^^/^^−^ MEFs following oxidative DNA damage. We found that, as a potential consequence of the increased levels of PNKP protein in *strap*^−^^/^^−^ MEFs, the cells are subsequently more resistant, in comparison to *strap*^+/+^ MEFs, to the killing effects of hydrogen peroxide (Figure 5D). Furthermore, when we overexpressed PNKP in *strap*^+/+^ MEFs to mimic the increased protein level observed in *strap*^−^^/^^−^ MEFs (Supplementary Figure S5), this also increased the resistance of the cells to hydrogen peroxide-induced cell death, suggesting that PNKP plays a major role in resistance to oxidative stress (Figure 5E).

Taken together, our data demonstrate that ATM-dependent phosphorylation of PNKP at serines 114 and 126 in response to oxidative DNA damage, inhibits its ubiquitylation-dependent degradation by the Cul4A-DDB1-STRAP ubiquitin ligase complex, resulting in the stabilization and accumulation of the PNKP protein and the increased ability to repair oxidative DNA damage.

## DISCUSSION

DNA damage generated endogenously as a result of cellular oxidative metabolism, can pose a major threat to genome integrity. DNA SSBs containing various modified 5′- and/or 3′-ends that arise as by-products of oxidative DNA damage repair need to be resolved to facilitate the repair process. PNKP plays a crucial role in this process by utilizing its associated DNA 5′-kinase and 3′-phosphatase activities to clean-up the DNA ends in preparation for DNA polymerase and DNA ligase functions. Indeed, the importance of PNKP in the cellular response to DNA damage has been highlighted by the increased sensitivity of PNKP shRNA knockdown cells to genotoxic agents (8) and that a deficiency in PNKP activity can result in human diseases (9). However, the mechanism(s) by which cells regulate the cellular levels of this important DNA repair protein were previously unknown. We now demonstrate that PNKP protein degradation is initiated by the Cul4A-DDB1-STRAP E3 ubiquitin ligase complex that monoubiquitylates PNKP at lysines 414, 417 and 484, at least *in vitro*, and thus initiates its proteasomal degradation. Although it is believed that monoubiquitylation is a signal for regulating the activity or cellular localization of the protein, rather than protein degradation, we have recently described that DNA polymerase β is also monoubiquitylated by the E3 ubiquitin ligase Mule, prior to polyubiquitylation by the ubiquitin ligase CHIP and subsequent proteasomal degradation (21,27). A similar mechanism may exist for PNKP whereby the protein is firstly monoubiquitylated by Cul4A-DDB1-STRAP, which can then act as a signal for polyubiquitylation and degradation. However, we did not observe any changes in the stability of PNKP following down-regulation of CHIP by siRNA (unpublished data), indicating that if this mechanism exists, then CHIP is not acting as the E4 ubiquitin ligase for PNKP and that another potential enzyme exists. We did, however, demonstrate that PNKP is substantially polyubiquitylated following its immunoprecipitation from *strap*^+/+^ cells, in comparison to *strap*^−^^/^^−^ cells, proving that polyubiquitylation of PNKP in cells is STRAP-dependent.

Cul4 is a member of the cullin family of proteins that acts as a scaffold for the assembly of multiple E3 ubiquitin ligase complexes (reviewed in (28)). The C-terminus of Cul4 interacts with the RING finger protein Roc1, which binds with the E2 conjugating enzyme, and the N-terminus of Cul4 interacts with DDB1. In turn, DDB1 acts as an adapter by further interacting with multiple WD-40 repeat proteins containing a conserved tandem repeat known as the DWD box, that provide specificity of ubiquitylation by interacting with the specific protein targeted for ubiquitylation (22–25). Currently, ∼100 WD-40 repeat proteins containing the DWD box have been identified, indicating that ∼100 different E3 ubiquitin ligase complexes exist that contain the Cul4-DDB1-Roc1 core component (22). We have now identified that STRAP, a previously identified WD-40 repeat protein (14,29), also contains the conserved DWD box that binds to DDB1. We further provide evidence that the Cul4A-DDB1-STRAP ubiquitin ligase complex exists in human cells, since the protein complex can be isolated from HeLa cells following multiple column chromatographies, and that the complex can be immunoprecipitated from cells overexpressing the individual components of the complex. Although we observed that the efficiency of complex formation between Cul4A-DDB1 and STRAP was relatively low (<1%), this can be explained by the fact that multiple (>100) ubiquitin ligase complexes can be formed from the Cul4A-DDB1 core subunits, which interact with the multiple WD-40 repeat proteins that provide specificity of ubiquitylation, of which STRAP is an example.

**Figure 6.**
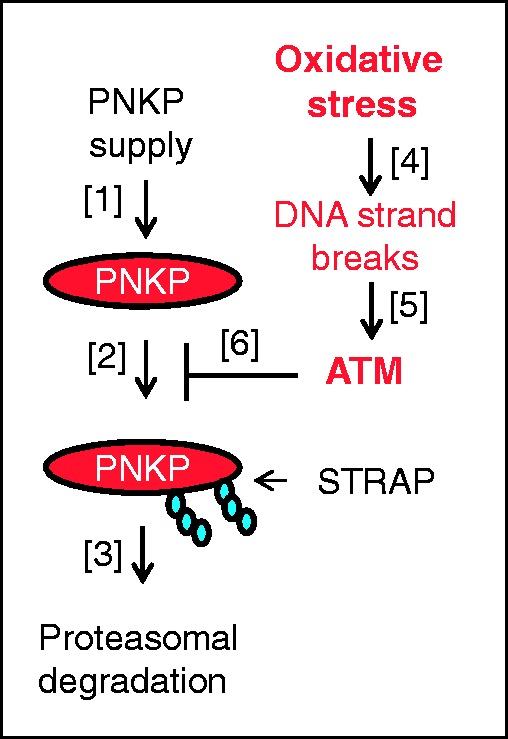
Proposed mechanism for the regulation of PNKP protein levels in response to oxidative DNA damage. The steady-state level of PNKP protein is determined by its generation through mRNA transcription and translation [1] and Cul4A-DDB1-STRAP-mediated ubiquitylation [2] which targets the protein for proteasomal degradation [3]. However, in response to oxidative stress [4], the generation of DNA strand breaks results in ATM activation [5] that consequently phosphorylates PNKP, which inhibits its ubiquitylation [6] and therefore its proteasomal degradation, and results in an elevation in PNKP protein levels that are required for DNA repair.

Phosphorylation of PNKP at serine 114 and 126 by ATM has recently been shown to occur in response to ionizing radiation and was required for effective DNA double-strand break repair (10,11). We now provide evidence that in response to oxidative DNA damage, ATM-dependent phosphorylation of PNKP at serine 114 and 126 also inhibits ubiquitylation-dependent degradation by the Cul4A-DDB1-STRAP ubiquitin ligase complex allowing PNKP protein levels to accumulate for effective DNA repair. This suggests that the protein levels of PNKP are finely tuned to the levels of DNA damage in the cell, as we have previously described for DNA polymerase β (27), and that only when excessive DNA damage is detected does the cell adjust the levels of PNKP that are necessary for DNA repair. Therefore, the cellular steady-state protein levels of PNKP are most probably a dynamic equilibrium between two processes: firstly PNKP supply is determined by the rate of *pnkp* gene transcription and corresponding mRNA translation (Figure 6, step 1), and secondly the rate of PNKP degradation is controlled by the Cul4A-DDB1-STRAP ubiquitin ligase complex (Figure 6, steps 2–3). Our findings also provide a molecular mechanism for enhancing cellular DNA repair capacity in response to acute oxidative stress. Since excessive oxidative DNA damage produces DNA double- and single-strand breaks, we assume that these breaks activate ATM that phosphorylates PNKP and thus prevents its ubiquitylation and degradation (Figure 6, steps 4–6). It is widely accepted that ATM deficiency is associated with sensitivity to oxidative stress (30) and we propose that under these conditions cells lacking functional ATM would not be able to enhance the required amount of PNKP protein. As a consequence, this will lead to a pathology associated with accumulation of DNA lesions and sensitivity to oxidative stress, and indeed ataxia-telangiectasia patients who have absent or inactive ATM protein display features of neurodegeneration, premature aging or genomic instability (30). Our data therefore provide new mechanistic insights into the phenotypes associated with a deficiency in ATM, one of the major proteins involved in the DNA damage response.

## SUPPLEMENTARY DATA

Supplementary Data are available at NAR Online: Supplementary Figures 1–5.

## FUNDING

Medical Research Council and Cancer Research UK (to G.L.D.); Biomedical Research Centre (NIHR), Oxford, UK (to B.M.K.); National Institute of Health/National Cancer Institute and the Veterans Administration (to P.K.D.). Funding for open access charge: Medical Research Council and Cancer Research UK.

*Conflict of interest statement*. None declared.

## Supplementary Material

Supplementary Data
