# Supplementary material for: Phosphorylation of PNKP by ATM prevents its proteasomal degradation and enhances resistance to oxidative stress
Source: Nucleic Acids Res. 2012 Oct 5;40(22):11404–15. doi: 10.1093/nar/gks909 (PMC3526271; doi:10.1093/nar/gks909)
Supplement: Supplementary Data [file supp_40_22_11404__index.html]

Phosphorylation of PNKP by ATM prevents its proteasomal degradation and enhances resistance to oxidative stress — Phosphorylation of PNKP by ATM prevents its proteasomal degradation and enhances resistance to oxidative stress — Supplementary Data 

# Phosphorylation of PNKP by ATM prevents its proteasomal degradation and enhances resistance to oxidative stress

## Supplementary Data

files

**Files in this Data Supplement:**

- Supplementary Data - docx file
