# Supplementary material for: Phosphorylation of PNKP by ATM prevents its proteasomal degradation and enhances resistance to oxidative stress
Source: Nucleic Acids Res. 2012 Oct 5;40(22):11404–15. doi: 10.1093/nar/gks909 (PMC3526271; doi:10.1093/nar/gks909)
Supplement: Supplementary Data [file supp_gks909_nar-01949-d-2012-File003.docx]

**SUPPLEMENTARY FIGURES**

**
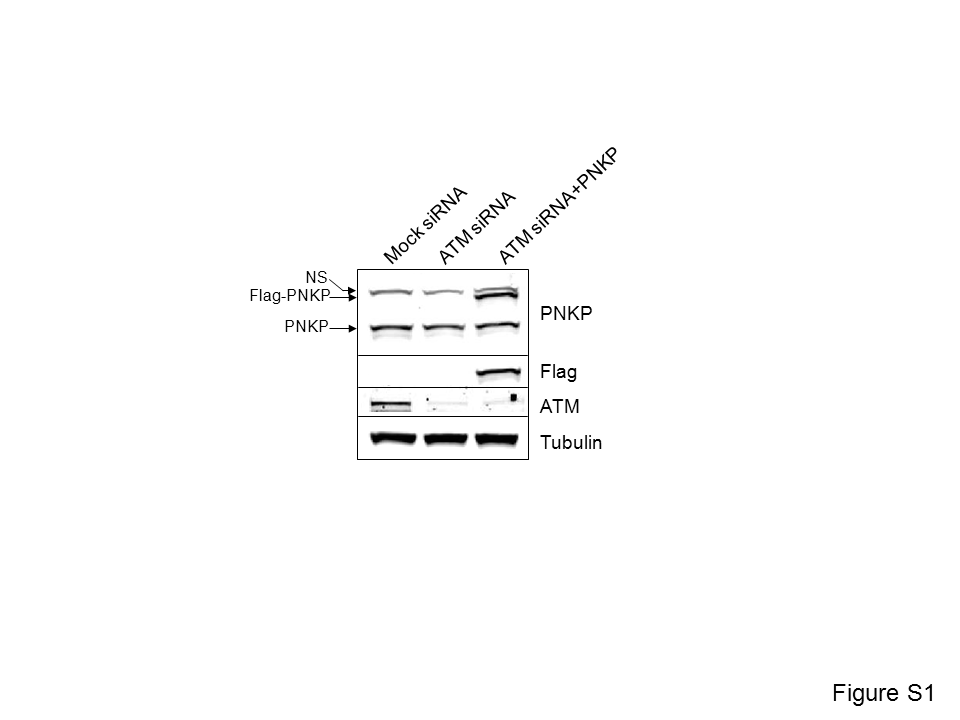
**

**Figure S1. Western blot of cell extracts prepared from HCT116p53^+/+^ cells treated in the absence and presence of ATM siRNA and a mammalian expression plasmid for PNKP.** HCT116p53^+/+^ cells were grown in 10 cm dishes for 24 h to 30-50 % confluency and then treated with Lipofectamine transfection reagent (10 µl) in the absence (Mock siRNA) or presence of 200 pmol ATM siRNA for 24 h. Cells were then treated with Lipofectamine transfection reagent (10 µl) in the absence (Mock siRNA and ATM siRNA) and presence (ATM siRNA+PNKP) of a mammalian expression plasmid expressing Flag-tagged PNKP (500 ng) for a further 24 h. Cells were pelleted by centrifugation, whole cell extracts were prepared and analysed by 8 % SDS-PAGE and immunoblotting with the antibodies indicated on the right hand side of the figure. The positions of endogenous (PNKP) and transfected (Flag-PNKP) protein are indicated on the left hand side of the figure and NS refers to a non-specific protein that cross-reacts with the PNKP antibodies.

**
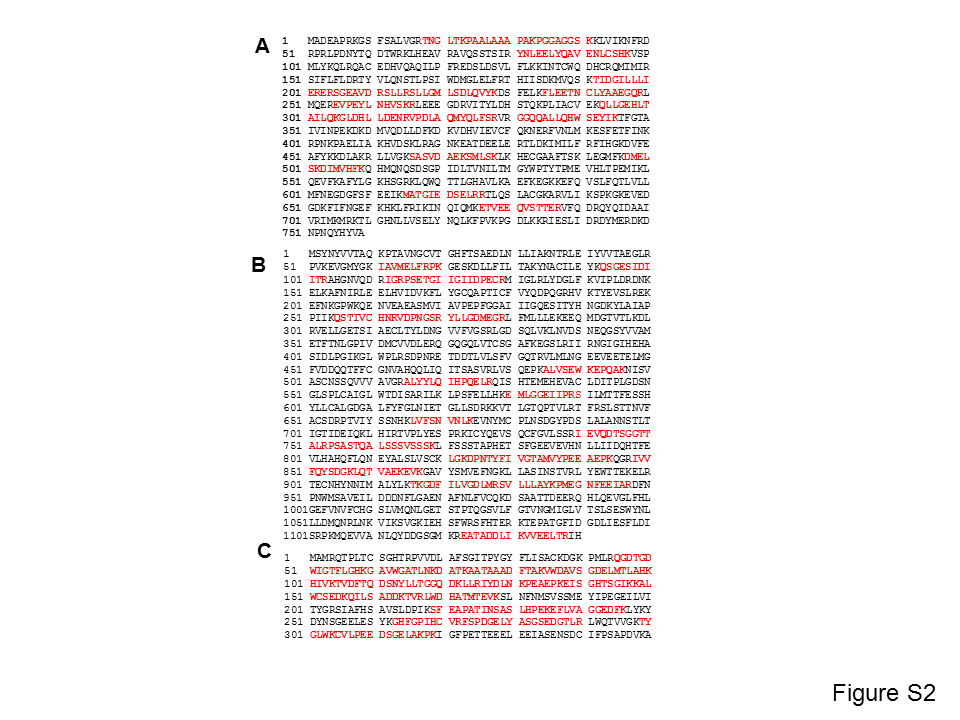
**

**Figure S2. Protein sequences of Cul4A, DDB1 and STRAP.** Protein sequence of (**A**) Cul4A, (**B**) DDB1 and (**C**) STRAP, with the peptide sequences detected by nanoLC-MS/MS from the final Mini-Q chromatography fractions purified from HeLa whole cell extracts highlighted in red.

**
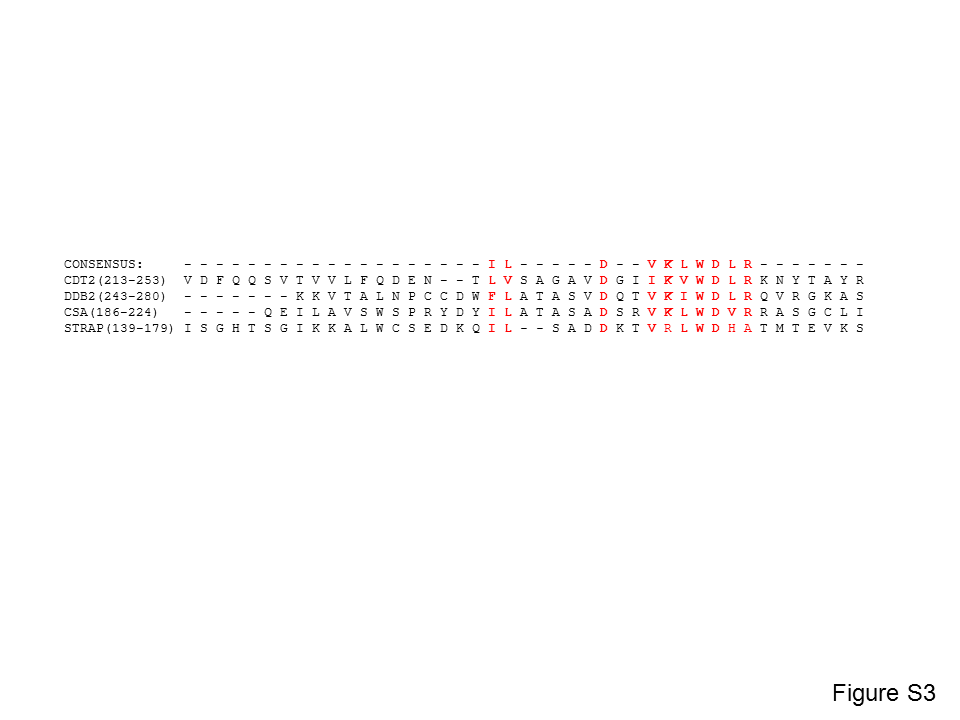
**

**Figure S3. Consensus protein sequence of the DWD box and the alignment with known DDB1-interacting proteins and STRAP.** The protein sequence of the conserved tandem repeat of DXXXR/KXWDXR/K, also known as the DWD box, which has been shown to bind DDB1 and is found in known DDB1-interacting proteins such as CDT2, DDB2 and CSA, is also found in STRAP. Conserved residues are highlighted in red.

**
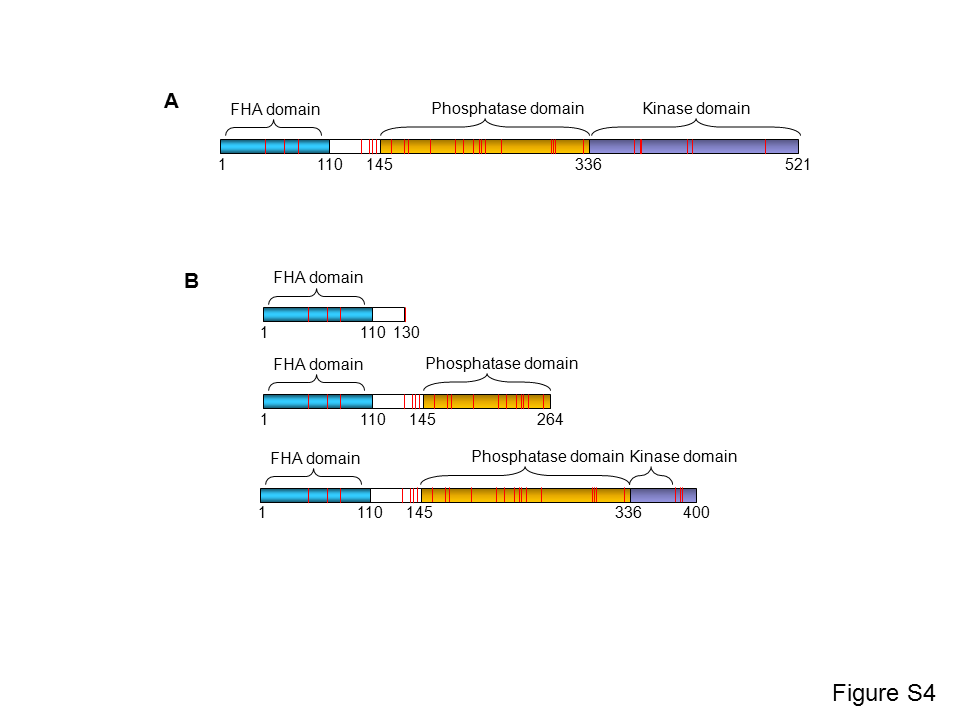
**

**Figure S4. Schematic diagram of the full length PNKP protein structure and also structures of truncated proteins used to identify the major sites of ubiquitylation.** (**A**) Schematic diagram of the full length protein structure of PNKP, highlighting the N-terminal forkhead-associated (FHA) domain, the central phosphatase domain and the C-terminal kinase domain. The positions of lysine residues within the protein structure that are potential ubiquitylation sites are indicated in red. (**B**) Truncated proteins fragments of PNKP were generated to identify the sites of ubiquitylation by Cul4A-DDB1-STRAP. These fragments contained the FHA domain (amino acids 1-130; top figure), the FHA and partial phosphatase domain (amino acids 1-264; middle figure) and the FHA, phosphatase and partial kinase domains (amino acids 1-400; bottom figure).

**
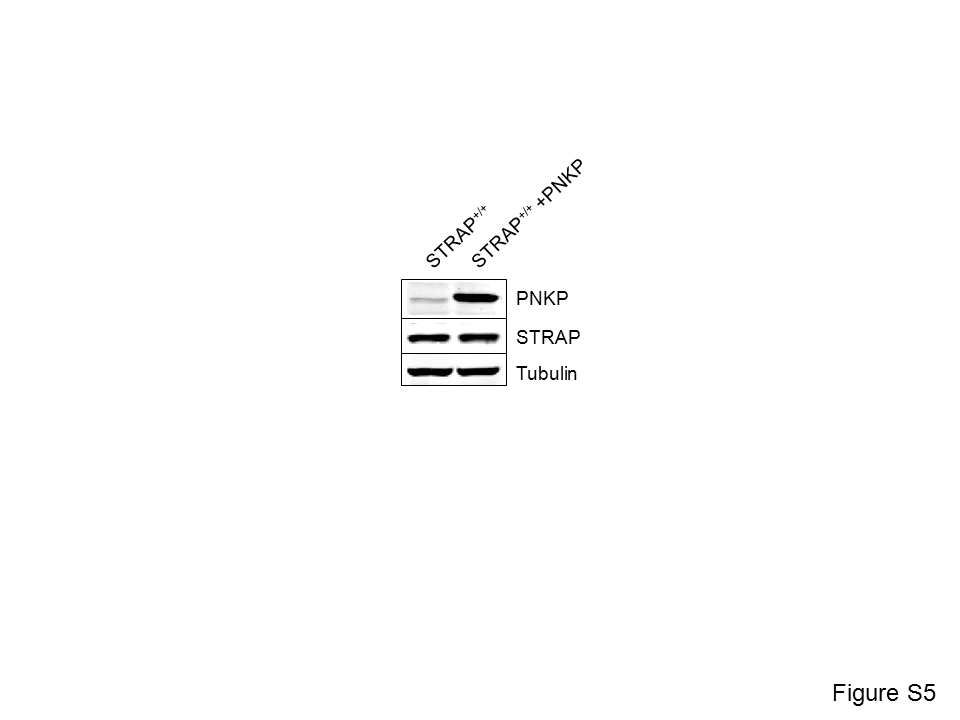
**

**Figure S5. Western blot of cell extracts prepared from *strap*^+/+^ cells treated in the absence and presence of a mammalian expression plasmid for PNKP.** *Strap*^+/+^ MEFs were grown in 10 cm dishes for 24 h to 80-90 % confluency and then treated with Lipofectamine (10 µl) in the absence and presence of a mammalian expression plasmid for PNKP (1 µg) for a further 24 h. Cells were pelleted by centrifugation, whole cell extracts were prepared and analysed by 8 % SDS-PAGE and immunoblotting with the antibodies indicated on the right hand side of the figure. Note that the observed level of overexpression of PNKP (approximately 20-fold) compared to endogenous protein is an overestimate, due to less cross-reactivity of the PNKP antibodies with mouse protein contained with the MEFs, in comparison to transfected human protein.
